# Perceived Reasons, Incentives, and Barriers to Physical Activity in Swedish Elderly Men

**DOI:** 10.2196/ijmr.3191

**Published:** 2014-11-12

**Authors:** Camilla Sjörs, Stephanie E Bonn, Ylva Trolle Lagerros, Arvid Sjölander, Katarina Bälter

**Affiliations:** ^1^Karolinska InstitutetDepartment of Medical Epidemiology and BiostatisticsStockholmSweden; ^2^Karolinska InstitutetDepartment of MedicineClinical Epidemiology UnitStockholmSweden

**Keywords:** Internet, exercise, motivation, barrier, aged, men

## Abstract

**Background:**

Knowledge about factors influencing physical activity behavior is needed in order to tailor physical activity interventions to the individual.

**Objective:**

The aim of this study was to explore and describe the perceived reasons, barriers, and incentives to increased physical activity, as well as preferable activities, among elderly men in Sweden.

**Methods:**

In total, 150 men aged 50-86 years responded to a Web-based questionnaire. Men who reported that they exercised sometimes or often received questions about reasons for physical activity (n=104), while men who reported that they never or seldom exercised received questions about barriers (n=46).

**Results:**

The most frequent perceived reason for being physically active was health (82%), followed by enjoyment (45%), and a desire to lose/maintain weight (27%). Lack of interest/motivation was identified as the primary perceived barrier (17%). Incentives for increasing the level of activity included becoming more motivated and having a training partner. Walking was the most preferred activity.

**Conclusions:**

Enjoyment and maintaining a good health were important reasons for engaging in physical activity among Swedish elderly men.

## Introduction

Levels of physical activity decrease with age. This tendency is unfortunate since an active lifestyle offers many health benefits such as improvement of weight control, reduced risk of cardiovascular disease, some cancers, type 2 diabetes, osteoporosis, and falls as well as reduced symptoms of depression [1-3]. The World Health Organization rates physical inactivity as one of the leading risk factors of death in the world [3]. Among Swedish men aged 45-84 years, 36% report they are physically active less than 30 minutes per day [4]. To reverse this negative trend, effective interventions promoting physical activity and preventing sedentary behavior in elderly are needed. Considering that men are under-represented in most physical activity intervention trials, there is a need to target this specific group [5,6].

Two previous review articles have summarized results from intervention studies on older adults [7,8]. One review suggested that an intervention program should include activities at a moderate intensity level that were convenient to engage in and reasonably inexpensive [7]. The other showed that the success of an intervention depended on motivation, social support, health, beliefs, and education of participants [8]. Therefore, when designing an intervention aimed at increasing individuals’ physical activity level, it is important to understand the reasons behind being physically active or not [9]. Physical activity interventions for older adults should include flexible programs tailored to the health status, preferences, and barriers of participants [7,8,10,11]. Among elderly in general, including both men and women, a wide range of barriers have been reported, including perceived poor health and physical disabilities, unsafe neighborhoods or no accessible physical activity environment, fear of injury, and lack of time [7,8,10,11]. Given that elderly highly respect their physician’s advice, health care professionals have a unique and underutilized possibility to encourage physical activity [7,8, 10,11]. A belief in health benefits is a common reason to adopt an active lifestyle, and in order to sustain the behavior, knowledge about health benefits needs to be accompanied with feelings of enjoyment [10]. However, few studies have assessed perceived reasons and barriers among elderly men specifically.

This study assessed perceived reasons for being physically active among active elderly men, and perceived barriers for being physically active in insufficiently active men. A similar approach has been used in previous studies [12-14], enabling a more direct assessment of perceived reasons and barriers in these specific groups. Sending out a questionnaire to prospective participants in the initiation phase of an intervention could be a feasible way to gain knowledge on perceived reasons, incentives and barriers in the target group. The aim of the present study was to explore the perceptions of reasons, incentives, and barriers to physical activity among Swedish elderly men, as well as their preferable activities.

## Methods

### Study Design

In September 2012, 1348 men who had agreed to be contacted for future substudies after having enrolled in an earlier cohort study [15], were invited to participate in this study via email, of which 31 had an invalid email address. In total, 167 men agreed to participate and attended a meeting at the study center. All participants were given oral and written information about the study and gave their written informed consent, and the questionnaire was then sent out via email. In total, 164 men answered the questionnaire, of which 5 did not respond to the question about perceived reasons/barriers and were excluded from further analysis. Additionally, 9 men younger than 50 years of age were excluded. In total, 150 men were included in the analysis. The study was approved by the Regional Ethics Committee at Karolinska Institutet, Stockholm, Sweden.

### Questionnaire

The questionnaire included short, structured questions, predefined answers as well as skip and follow-up patterns. Individual user names and passwords served as identifiers. Year of birth, height, weight, education, and current occupation were self-reported in the questionnaire. The questions about perceived reasons and barriers were identified from previously validated scales, including the Decisional Balance measure for exercise (DB) [16,17], the Exercise Benefits/Barriers Scale (EBBS) [18,19] and the Motivation for Physical Activity Measure (MPAM) [20,21]. In addition, we also included statements on rehabilitation and fear of injury. All statements were modified and combined in order for the questions to suit a Swedish population and contained a maximum of 10 statements in total to increase the user-friendliness and be compatible with the web design of the questionnaire.

Participants were initially asked to select an activity level and were categorized as physically active if they answered that they exercised *occasionally* or *often* and insufficiently active if they *never* or *seldom* exercised. Men answering *none of the alternatives apply to me* or that they *cannot exercise due to illness* did not receive a follow-up question about perceived reasons/barriers and were excluded. The physically active men received a follow-up question with ten predefined statements regarding reasons for exercising, and the insufficiently active men received a follow-up question with nine statements regarding barriers. Participants were asked to indicate on a 5-point scale to what extent they agreed to each statement. The statements were presented in random order to each respondent.

All participants responded to a question about incentives for increasing physical activity. They were asked to select all response alternatives that applied to them from eight predefined statements, or if they thought they were physically active enough, or did not know. All men (except those responding to being physically active enough) received a follow-up question where they selected which specific activities they would prefer to do from eight predefined alternatives.

### Statistical Analysis

Descriptive results are presented as means and standard deviations (SD) or numbers of participants (n) and percentages. To assess potential differences between physically active and insufficiently active participants, *t* test was used on continuous variables (age, weight, height, body mass index) and Fisher’s exact test on categorical variables (occupation, education, body mass index, and incentives for increasing physical activity). These tests were used because they do not rely on asymptotic theory, which is an important advantage given our small sample size. Fisher’s exact test was also used to assess potential differences between younger and older men regarding perceived reasons and barriers. The significance level was set to alpha=.05. Analyses were performed using STATA 12 (STATA Corporation, College Station, TX, USA).

## Results

### Characteristics of Participants

Characteristics of the study participants are presented in Table 1. The mean age was 66.6 years (range 50 to 86). Almost 70% of the participants were physically active and received a follow-up question about perceived reasons to physical activity. The remaining 30% were classified as insufficiently active and received a follow-up question about perceived barriers.

### Perceived Reasons for Exercise

**Figure 1:**
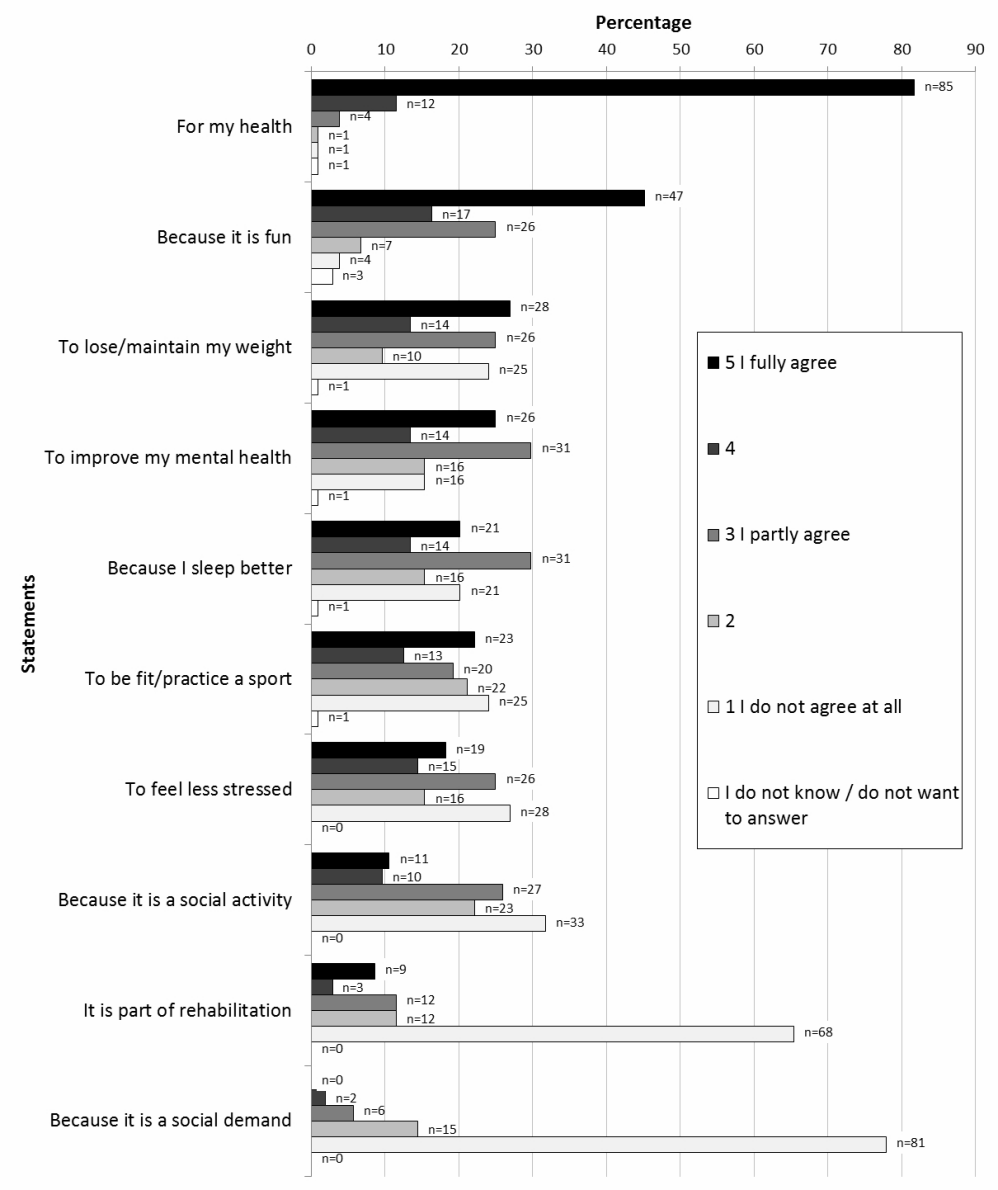
Identification of different perceived reasons for activity among physically active men (n=104).

Health factors was the most frequent perceived reason for physical activity (82%), followed by enjoyment (45%) and a desire to lose/maintain weight (27%) (Figure 1). There was a statistically significant difference between men younger than 65 years and men older than 65 years with regards to reporting of health and enjoyment as reasons. Younger men (aged 50-65) reported health factors and reducing stress levels more often than older men who reported enjoyment to a greater extent.

**Table 1:** Characteristics of the study population.

Characteristics		All participants (n=150)		Physically active (n=104)		Insufficiently active (n=46)		*P*
		Mean (SD) or n (%)		Mean (SD) or n (%)		Mean (SD) or n (%)		
Age (years)		66.6	(7.5)	65.7	(7.0)	68.6	(8.2)	.03
Weight (kg)		82.4	(10.6)	81.3	(9.2)	84.8	(12.9)	.05
Height (cm)		179.1	(6.3)	179.3	(6.3)	178.6	(6.6)	.51
Body mass index (kg/m^2^)		25.6	(2.8)	25.3	(2.6)	26.5	(3.0)	.011
**Body mass index,kg/m** ^**2**^								.012
	<25 (normal weight)	63	(42.0)	51	(49.0)	12	(26.1)	
	≥25 (overweight and obese)	87	(58.0)	53	(51.0)	34	(73.9)	
**Occupation**								.21
	Work	84	(56.0)	62	(59.6)	22	(47.8)	
	Do not work/retired	66	(44.0)	42	(40.4)	24	(52.2)	
**Education, years**								.19
	≤ 9	24	(16.0)	14	(13.5)	10	(21.7)	
	>9-12	43	(28.7)	29	(27.9)	14	(30.4)	
	>12	70	(46.7)	49	(47.1)	21	(45.7)	
	Other	13	(8.7)	12	(11.5)	1	(2.2)	

### Perceived Barriers for Exercise

**Figure 2:**
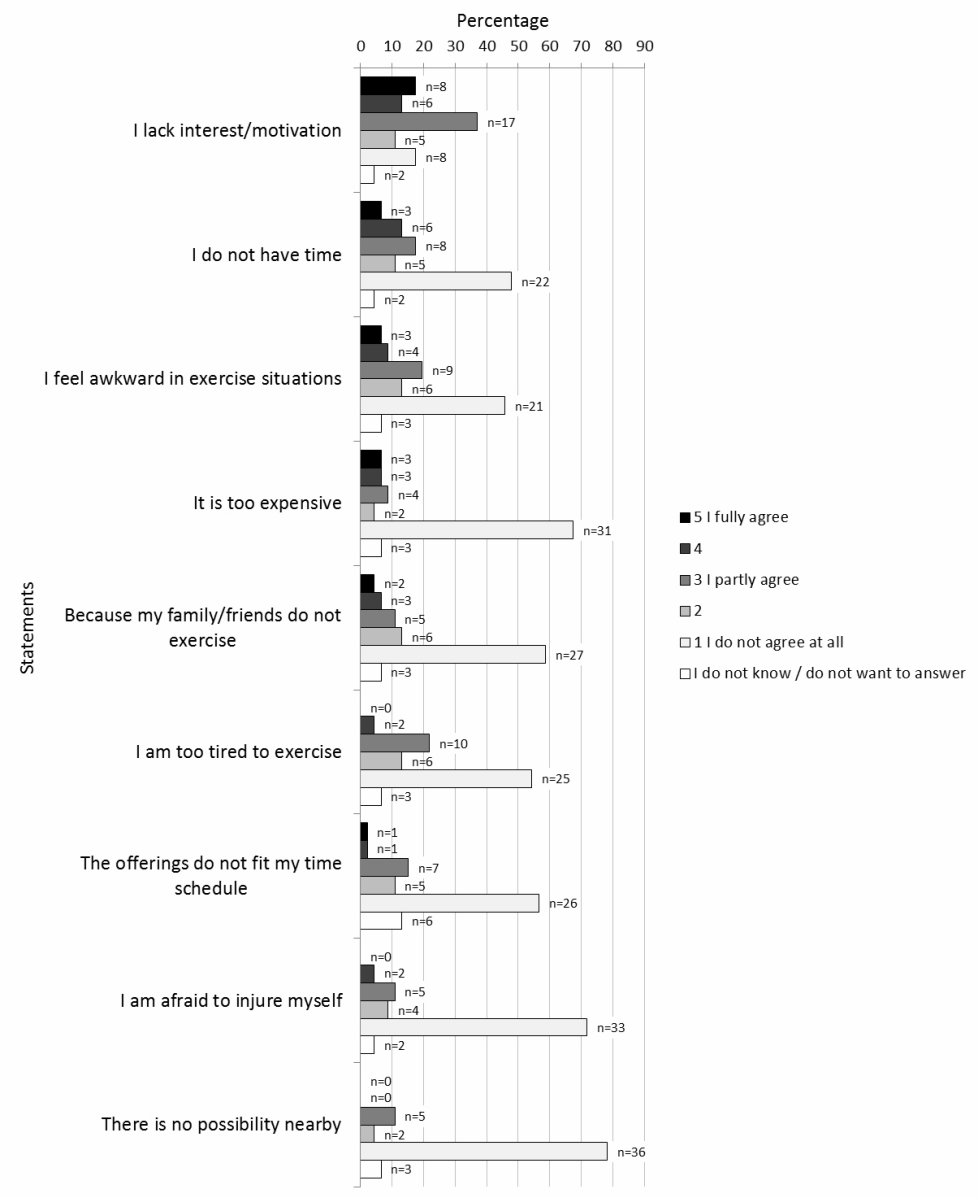
Identification of different perceived barriers to physical activity among insufficiently active men (n=46).

Lack of interest/motivation was the most frequent perceived barrier, fully agreed by 17% of the insufficiently active men (Figure 2), followed by lack of time and feeling awkward. The majority of the insufficiently active participants did not agree with any of the remaining barriers. We did not detect any clear patterns when testing for differences between younger and older men with regards to perceived barriers of physical activity.

**Figure 3:**
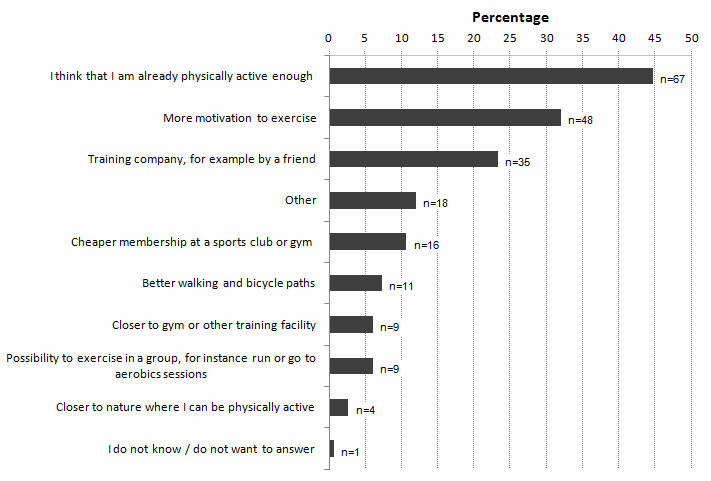
Incentives that would make the participants more physically active (n=150). The 18 men answering “Other” received a follow-up question with the possibility to write down what would make them more physically active. Eight of the men answered less pain during exercise and six needed more leisure time.

Nearly half of the men, 45%, reported that they thought they were physically active enough (48% of the active and 37% of the insufficiently active men). Incentives for increasing physical activity among the remaining 55% included becoming more motivated, having a training partner, less expensive memberships at health clubs, and improved paths for walking or biking. Insufficiently active men reported becoming more motivated and having a training partner to a greater extent than physically active men (50 vs 24%, and 35 vs 18%, respectively) (Figure 3).

**Figure 4:**
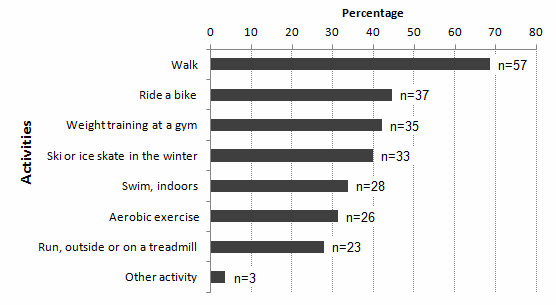
Preferred activities among men that would like to be more physically active (n=83).

Men wanting to be more physically active received a follow-up question about preferred activities (Figure 4). The most preferred activities were walking, biking, weight training and skiing/skating.

## Discussion

### Principal Findings

Health and enjoyment were the main perceived reasons for maintaining a physically active lifestyle among the elderly men in this study, while lack of interest was the most common perceived barrier. Being more motivated and having a training partner were considered factors that would increase the level of physical activity.

### Perceived Reasons for Exercise

We found that maintaining good health is the main perceived reason for being physically active which is in line with results from previous studies of middle-aged and older men and women [1,13,22,23]. Staying physically independent in daily life has commonly been shown to be important for elderly [24-26]. One could speculate that some of the participants in the present study had this in mind when rating high agreement with the health statement.

In contrast to our findings, several studies report social reasons to be the most important [27,28] or second most important [1,13] reason for middle-aged and older men and women encouraging physical activity. However, men were under-represented in most of the studies mentioned above. Only a small proportion of the physically active men in our study reported being active for social reasons. Nevertheless, our results indicated that training partners are an important incentive when encouraging the insufficiently active men to become more physically active.

Enjoying exercise was the second most frequent perceived reason for activity in our study, similar to results from a previous study of elderly Iranian men and women [28]. Lifelong participation and enjoyment of physical activity were one of the main reasons in a review of motivational factors for increased activity level after retirement [23]. According to another overview, motives of fun were more likely to be reported by younger individuals than older adults [26]. Nonetheless, our results add to the number of studies showing the crucial role of enjoyment for physical activity adherence and maintenance [12,20,29,30]. Further, weight management was a statement highly agreed with in this study, which has also been seen previously [22,24]. In order to maintain the activity needed for weight loss, it is important that overweight men find activities they enjoy [20,30].

### Perceived Barriers for Exercise

We found that lack of interest/motivation was the main barrier to exercise which is in agreement with results from previous studies [31,32]. In contrast, Smith et al [33] did not find a relation between lack of motivation and nonparticipation in physical activity among healthy Canadian men above the age of 60 years. As almost 90% of the participating men did not identify any barrier, the authors concluded that barriers were not the limiting factor for absence of physical activity. Rather, they speculate that the cause for inactivity may instead have been underlying chronic health conditions.

Half of the men in our study did not perceive lack of time as a barrier to physical activity. Previous studies on the matter have been conflicting. While some studies have reported that time barriers decrease upon aging [11,26,34], a number of studies have found time to be the main barrier for both middle-aged and older men and women [13,23,35,36].

Overall, a majority of the insufficiently active men did not identify themselves with the predefined barrier statements, indicating there were other barriers of importance not included in the questionnaire. For example, a vast number of studies have claimed that health problems are the main barrier for engagement in physical activity among elderly [1,14,25,31]. Those men who were not able to be physically active due to illness were excluded in this study. Nevertheless, in retrospect, inclusion of a statement about health problems would have been appropriate. Another explanation could be the fact that one-third of the men categorized as insufficiently active thought that they already had enough physical activity. The finding could also be due to low awareness of underlying barriers among participants. Interestingly, several previous studies have reported that participants did not identify any, or only a few, barriers for engagement in physical activity [1,12,24,33].

### Strengths and Limitations

There are further limitations and strengths that merit discussion. Our findings should be interpreted with caution, given that we have carried out a relatively large number of tests. We have chosen not to make explicit corrections for multiple tests; since those corrections are not uncontroversial [37]. Data for the present study were collected in a group of elderly men living in Stockholm and volunteering to be part of the study, likely being more health conscious than the rest of the population. Almost 50% of our participants had an education longer than 12 years compared to 27% in the general Swedish population of elderly men [38]. In addition, 58% were overweight/obese compared to 65% of Swedish men aged 55-74 years in general [39]. As a result, it is not possible to extrapolate the results. Although we derived our statements about perceived reasons and barriers from established scales, we did not validate them in our population. It should be highlighted that the stated reasons for being active are perceived motivational constructs, and not necessarily what is truly regulating the men’s behavior. We cannot rule out that important reasons and barriers may have been missed in the survey. Nonetheless, the Internet allowed both flexibility and privacy for the participants and provided a quick and convenient way to assess information about the target group.

### Practical Implications

The success of an intervention depends on a wide variety of factors affecting physical activity behavior [7,8,10,11]; therefore, interventions among elderly men should be tailored to each individual. Given that elderly are positive towards receiving advice from health care professionals [10], physicians, physiotherapists, and nurses could play a key role in encouraging an active lifestyle, as is done in Sweden where Physical activity on prescription is used [40,41]. Assessing reasons, incentives, and barriers for physical activity may be useful both for health care professionals and during an intervention, to individually tailor support. However, a major challenge is that many insufficiently active older adults overestimate their physical activity behavior and believe they are active enough [10,42]. These participants need to be made aware of the insufficiency of their current activity level to respond effectively to the intervention [42]. A tailored program should include a variety of physical activity options, including activities performed with training partners. Already active participants could receive help in goal setting, monitoring of progress, and self-reinforcement, which increases the likelihood of sustained behavior [10].

### Conclusions

In the group of elderly men in Sweden who participated in this study, the main reasons for being physically active were enjoyment and maintaining good health. Incentives for increasing physical activity included having a training partner, and lack of interest/motivation was identified as the primary barrier. However, reasons, incentives, and barriers for physical activity differed significantly among the participants. As a result, we encourage the assessment of these ahead of an intervention study to enable individual tailoring.

## Abbreviations

DB: Decisional Balance
EBBS: Exercise Benefits/Barriers Scale
MPAM: Motivation for Physical Activity Measure
